# Redefining the Relationship: Palliative Care in Critical Perinatal and Neonatal Cardiac Patients

**DOI:** 10.3390/children8070548

**Published:** 2021-06-25

**Authors:** Natasha S. Afonso, Margaret R. Ninemire, Sharada H. Gowda, Jaime L. Jump, Regina L. Lantin-Hermoso, Karen E. Johnson, Kriti Puri, Kyle D. Hope, Erin Kritz, Barbara-Jo Achuff, Lindsey Gurganious, Priya N. Bhat

**Affiliations:** 1 Sections of Critical Care Medicine and Cardiology, Department of Pediatrics, Texas Children’s Hospital and Baylor College of Medicine, Houston, TX 77030, USA; afonso@bcm.edu (N.S.A.); Rachel.Ninemire@bcm.edu (M.R.N.); jaime.jump@bcm.edu (J.L.J.); kriti.puri@bcm.edu (K.P.); erin.kritz@bcm.edu (E.K.); Barbara-Jo.Achuff@bcm.edu (B.-J.A.); 2 Section of Neonatology, Department of Pediatrics, Texas Children’s Hospital and Baylor College of Medicine, Houston, TX 77030, USA; sharada.gowda@bcm.edu (S.H.G.); karenj@bcm.edu (K.E.J.); 3 Section of Palliative Care Medicine, Department of Pediatrics, Texas Children’s Hospital and Baylor College of Medicine, Houston, TX 77030, USA; gurganio@bcm.edu; 4 Section of Cardiology, Department of Pediatrics, Texas Children’s Hospital and Baylor College of Medicine, Houston, TX 77030, USA; mrlanti2@texaschildrens.org (R.L.L.-H.); kyle.hope@bcm.edu (K.D.H.)

**Keywords:** palliative care, congenital heart disease, neonatology, perinatology

## Abstract

Patients with perinatal and neonatal congenital heart disease (CHD) represent a unique population with higher morbidity and mortality compared to other neonatal patient groups. Despite an overall improvement in long-term survival, they often require chronic care of complex medical illnesses after hospital discharge, placing a high burden of responsibility on their families. Emerging literature reflects high levels of depression and anxiety which plague parents, starting as early as the time of prenatal diagnosis. In the current era of the global COVID-19 pandemic, the additive nature of significant stressors for both medical providers and families can have catastrophic consequences on communication and coping. Due to the high prognostic uncertainty of CHD, data suggests that early pediatric palliative care (PC) consultation may improve shared decision-making, communication, and coping, while minimizing unnecessary medical interventions. However, barriers to pediatric PC persist largely due to the perception that PC consultation is indicative of “giving up.” This review serves to highlight the evolving landscape of perinatal and neonatal CHD and the need for earlier and longitudinal integration of pediatric PC in order to provide high-quality, interdisciplinary care to patients and families.

## 1. Introduction

*“The physician’s duty is not to stave off death or return patients to their old lives, but to take into our arms a patient and family whose lives have disintegrated and work until they can stand back up and face, and make sense of, their own existence.”*

*—Paul Kalanithi. When Breath Becomes Air*

### 1.1. Epidemiology of CHD

Congenital heart disease (CHD) is the most common birth defect and a leading cause of infant death regardless of gestational age, occurring in approximately 1 per 100 live births [1,2]. Of these patients, approximately 1% have hypoplastic left heart syndrome (HLHS), which is associated with the highest mortality among CHD patients [1]. Over the last several decades, advances in the prenatal, surgical, and post-operative care of CHD patients have drastically increased survival and life expectancy. However, a recent study evaluating 25-year survival outcomes of over 35,000 children undergoing cardiac surgery in the United States found that long-term survival was still decreased compared to healthy children for all forms of CHD, including the mildest lesions [3].

Among non-survivors, most deaths occur in intensive care units (ICU) after discontinuation of life-sustaining therapies following prolonged hospitalizations, that are often characterized by multi-organ dysfunction, mechanical circulatory support, and other invasive procedures [4]. Furthermore, even among long-term survivors, both patients and their families bear the burden of lifelong complications that can include repeat surgical interventions, cognitive deficits, progressive heart failure, and poor functional status [5]. Emerging literature indicates that the use of palliative care (PC) services in the prenatal, neonatal, and pediatric cardiac population is associated with greater use of comfort care at the end of life, fewer medical procedures, fewer deaths and hospital days in the ICU, decreased maternal anxiety, and improved family communication [6,7,8,9,10,11]. Therefore, pediatric PC programs have a role in supporting families not only at the end of life but also throughout their disease course, starting at the time of diagnosis.

### 1.2. CHD and the Need for Pediatric PC Engagement

Optimal pediatric PC includes care for both the child and family [12,13,14]. Several features characterize the unique and profound need for PC in CHD patients. First, 10–70% of CHD in the United States is diagnosed prenatally [14]. It has been reported that even at the time of initial diagnosis, parents of children with CHD experience higher levels of depression and anxiety compared to parents with healthy children or those with other chronic illnesses [6,15,16,17,18].

Second, optimal care of children with CHD requires a highly specialized multidisciplinary approach, including cardiology, critical care, congenital cardiac surgery, obstetrics, neonatology, anesthesiology, and nursing. Care is further augmented through services provided by social work, respiratory therapy, speech and physical therapy, music and art therapy, spiritual care, etc. Though well-meaning, the involvement of multiple teams can transmit subtle differences in opinion and messaging, which creates conflict and exacerbates the myriad of stressors faced by CHD families [16,17,19].

Third, prognostic uncertainty characterizes many types of CHD diagnoses and is compounded by the co-existence of CHD with genetic and/or additional congenital anomalies. CHD may be accompanied by heart failure, which can have a waxing and waning course, and the point beyond which survival is unlikely is often unclear. Children with single-ventricle physiology are particularly fragile and have a reported 6-year transplant-free survival of only 59% to 64%, despite undergoing multiple invasive procedures early in life. Complex medical decisions often have to be made early in a child’s life, including prenatally, when parents may need to decide whether or not to continue pregnancy [20,21,22].

Finally, although technological advances have improved survival in CHD patients, there have been concurrent increases in the responsibilities placed upon families to provide complex and chronic medical care at home and experience a high frequency of hospital readmissions [23]. While some of the features listed above may apply to patients with other diseases, their co-existence is unique to the CHD patient population. Pediatric PC has had demonstrable benefits in other pediatric subspecialities, but usage remains low within pediatric cardiology despite the high level of need and potential benefit [9,10,11]. The combination of all the factors listed above underscores the vast need for and important role that pediatric PC programs can play when supporting cardiac patients and family during challenging and uncertain times.

### 1.3. Objectives

In this review, we aim to describe the evolving landscape of neonatal and perinatal CHD, highlighting the many significant stressors faced by families, starting at the time of diagnosis and persisting throughout the course of the child’s illness. We present models for the early integration of pediatric PC services into multi-disciplinary care strategies for this unique patient population. We spotlight high-risk neonatal CHD patient groups: preterm infants and those with significant genetic anomalies, as these patients often have higher morbidity and mortality, and decision-making is particularly challenging. We describe how technological advances have improved survival in infants and children with CHD but have placed new burdens on families and medical providers, and how these may be mitigated by PC engagement. In societies that are more multi-cultural than ever, we discuss the need for inclusive and respectful PC for patients and families from all backgrounds. We discuss barriers to PC consultation in the vulnerable CHD patient population and propose solutions. Finally, we examine the novel burdens placed on parents of infants with CHD during the COVID-19 pandemic and the increased need for pediatric PC support.

## 2. Discussion

*“Death is not the opposite of life, but a part of it.”*

*—Haruki Murakami. Norwegian Wood*

In 2013, the American Academy of Pediatrics (AAP) Section on Hospice and Palliative Medicine and Committee on Hospital Care endorsed integration of PC teams in the longitudinal care of infants and children upon diagnosis of potentially life-limiting illnesses, regardless of the expected outcome [24]. Similarly, in 2017, the National Pediatric Cardiology Quality Improvement Collaborative (NPCQIC) recommended PC consultation for all infants with single ventricle CHD [7]. The number of PC programs for adults and children has grown steadily over the past decade, as routine PC consultation is more accepted as part of the multidisciplinary care that families receive starting at the time of diagnosis [25].

### 2.1. Prenatal Diagnosis of CHD and Maternal Stress

The prevalence of prenatal diagnoses of CHD has led to a greater understanding of the vulnerability of this unique patient group, starting in utero. Elevated psychobiological markers of maternal stress during pregnancy, especially early in gestation, may correlate with altered placental function, delayed fetal maturation, and decreased cerebellar and hippocampal volumes in fetuses with CHD [26]. In addition, these markers may be associated with disrupted emotional regulation and impaired cognitive performance during infancy, and decreased brain volume in areas associated with learning and memory in 6- to 8-year-old children [27]. PC involvement even before delivery may significantly mitigate maternal stress, which can affect long-term patient outcomes, though further studies are needed [6].

A review of our institutional Perinatal Pediatric Advanced Care Team (PPACT) database demonstrates that among fetuses with certain types of CHD that were deemed likely non-survivors and referred for PC consultation, roughly one-third did not survive to birth, and those that survived pregnancy expired in the early neonatal period. Fetuses diagnosed with lethal syndromes, complete heart block with hydrops fetalis or complex heterotaxy syndromes, obstructed total anomalous pulmonary venous connection, advanced hyrops fetalis secondary to other non-cardiac conditions, and HLHS with restrictive atrial septum, were particularly prone to poor outcomes. Fetal echocardiography was a reliable predictor of lethality and may serve as a valuable tool for the identification of pregnancies that may benefit from early PC consultation in the perinatal period [28].

### 2.2. Models for Integration of Pediatric PC into Perinatal Cardiology

**Figure 1:**
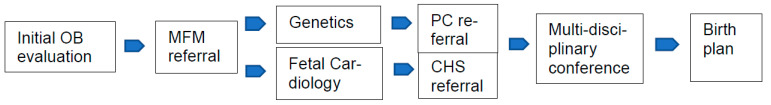
Proposed model for integration of a PC team into the care of high-risk fetal CHD diagnoses [28]. OB = obstetrics, MFM = maternal–fetal medicine, PC = palliative care, CHS = congenital heart surgery.

Perinatal PC is a coordinated care strategy that traverses obstetric and neonatal care by providing a safe, compassionate, and caring space for parents to process their emotions and discuss their values. In a shared decision-making model, the team explores how the family’s goals, values, and hopes in the setting of the current medical diagnosis and prognosis can help shape the landscape of overall care for their child. Available options following a CHD diagnosis are vast, although their breadth can vary in different parts of the world due to legal, economic, and cultural considerations. Choices may range from termination of the pregnancy in permissive societies to aggressive and invasive pre- and post-natal medical therapies, and all of these carry with them significant emotional, moral, financial, and physical burdens that could be mitigated by PC involvement. In certain scenarios, with the early engagement of PC, parents may initially choose to pursue a trial of interventional therapy for their newborn but switch goals of care seamlessly if needed based on their child’s evolving clinical condition and prognosis. Advocacy for maximizing quality of life and comfort for neonates diagnosed with CHD is quintessential in the current era of evolving surgical and hybrid techniques [29,30]. As described in Figure 1, prenatal consultation with pediatric PC, neonatology, cardiology, and maternal–fetal medicine along with risk-adjusted care in the delivery room will promote trust and improve perinatal care in neonates with complex CHD [28,31].

*“Remember it always. Remember that you and I made this journey and went together to a place where there was nowhere left to go.”*

*—Jhumpa Lahiri. The Namesake*

Building on existing data from adults with heart failure, Neubauer et al. identified six common psychological, emotional, and cognitive transitions in families after a life-limiting pediatric cardiac diagnosis [12]. Clearly, parents are at risk for experiencing intense and complex psychosocial stress as early as the prenatal period, and remain vulnerable through surgery and beyond [12,16,17]. Knowledge of these transitions can allow pediatric PC providers to address a range of issues and adequately support cardiac patients and families during these challenging times, as described in Table 1 [12].

### 2.3. High-Risk Subsets of Neonates with CHD: Prematurity

As pediatric PC becomes an integral part of providing balanced care to the cardiac population, certain subgroups such as preterm infants and those with pre- or post-natal diagnosed genetic anomalies may see additional benefits. One in six infants with cardiovascular malformations is born preterm, and the combination of CHD and prematurity often portends greater morbidity and mortality [32]. Delayed transition from fetal to neonatal circulation results in abnormal hemodynamics and may create a particularly vulnerable period for preterm infants with CHD. Common complications of prematurity, including respiratory distress syndrome, intrauterine growth restriction, neurologic injury, and necrotizing enterocolitis, may occur more frequently in infants with CHD, potentially altering the timing (or even the utility) of surgical intervention [33]. Early PC consultation can result in greater support for families facing prognostic uncertainty and challenging decision-making for their newborn.

### 2.4. High-Risk Subsets of Neonates with CHD: Genetic Anomalies

One study of six hospital-based pediatric PC programs identified that of 515 PC patients, the primary clinical problem was genetic or congenital in over 40%. In this cohort of patients, two-thirds were still alive following a 12-month follow-up period [34]. At many centers, genetic testing is routinely performed for infants diagnosed with CHD as cardiac anomalies are often associated with genetic conditions, such as Trisomy 21 and chromosome q22.11 microdeletion, as well as other anatomic and neurodevelopmental findings. However, the clinical significance of many genetic anomalies is not always known, and assisting families with medical decision-making or advanced care planning can be challenging. Identification of long-term goals or quality-of-life measures for the child can be instrumental in allowing parents to make informed decisions surrounding the care of their infants.

Over the last several decades, the types of cardiac surgical procedures that are offered to patients with specific genetic syndromes have changed. Historically, the accepted approach in this group was a palliative pathway focused on comfort care at home and avoidance of invasive surgical interventions, as these were typically associated with prolonged hospitalizations and poor outcomes. More recently, as outcomes may be improving, surgical repair is considered for some infants with severe chromosomal abnormalities, such as Trisomy 13 and Trisomy 18, both of which are associated with severe neurodevelopmental delays and a reported one-year survival rate of 10% [35,36]. Trisomy 18 is also associated with cardiac anomalies in 90% of patients, with the majority being uncomplicated defects, such as atrial septal defects, ventricular septal defects, and patent ductus arteriosus. Such defects can be repaired with a single operation and result in improved symptoms in the majority of patients [37,38]. However, the data on surgical outcomes of these patients is limited and may be difficult to interpret due to selection bias, as those patients who are offered surgical intervention may have less severe features [37]. In a recent analysis of a large surgical database, infants with Trisomy 13 and 18 were shown to have higher operative mortality than other children undergoing the same operation [39].

It is important to identify and appropriately counsel families of these infants, individualizing care based on clinical presentation. Infants with significant cardiac disease may not be surgical candidates, whereas those with less complex CHD may be considered. In the latter group of patients, surgical interventions may lessen heart failure symptoms and optimize time at home. As congenital heart surgery advances and pushes the boundaries of what cardiac procedures can be safely and realistically offered to infants with complex medical and genetic conditions, consideration must be given to ensuring that appropriate support systems are in place for families as they make pre- and post-natal decisions for their infant.

### 2.5. Technological Advances, Burdens, and Challenging Decisions in Infant Survivors with CHD

With the increasing use of life-prolonging technology in CHD patients, such as extracorporeal life support (ECMO), ventricular assist devices (VAD), and tracheostomies with home ventilators, families are often faced with difficult decisions regarding their child’s plan of care. Although pediatric PC providers are frequently involved in crises and helping to manage end-of-life issues, it is also important to note that the pediatric PC team can assist with decision-making support throughout the continuum of care [8]. This can include decisions to pursue additional surgical or catheter-based interventions, tracheostomy with the need for a home ventilator, gastrostomy tube, or other life-prolonging technology. Ideally, pediatric PC providers can help to ensure that invasive interventions are aligned with the goals of the patient and family as part of the longitudinal care team using shared decision-making. A recent study demonstrated that programmatic integration of PC in the care of pediatric patients on VAD support resulted in more documented advanced directives and compassionate withdrawal of mechanical support at the time of death [40].

### 2.6. Provision of Optimal Pediatric PC Services in a Multi-Cultural, Global Society

*“‘Tell us please, what treatment in an emergency is administered by ear?’...I met his gaze and I did not blink. ‘Words of comfort,’ I said to my father.”*

*—Abraham Varghese. Cutting for Stone*

To provide optimal end-of-life care for infants, children, and their families, PC providers need to have cultural humility and awareness for families from diverse backgrounds. Studies exploring cultural variations in end-of-life care in the pediatric cardiac ICU are limited [41]. Davies et al. reported that nearly 40% of healthcare providers identified cultural differences as a common barrier to adequate pediatric PC consultation [42]. Language differences can also render parents unable to adequately comprehend the information being shared or communicate questions and concerns effectively [43]. Faith and spirituality are known to play key roles in the ability of parents to cope with and make decisions surrounding their child’s condition [44]. Language, spiritual, and cultural differences can thus challenge the ability of healthcare providers to provide adequate information to parents about their child’s condition, treatment, and prognosis [42,43,44]. We support the growing involvement of pastoral workers in pediatric PC teams in order to provide spiritual support to families of diverse faiths and religious traditions during the course of a child’s life and in the event of death. More studies are needed to proactively address cultural, spiritual, and linguistic gaps in pediatric PC support for families of these infants and children so that a difficult experience is not made worse by inaccurate or inadequate communication.

The benefits of pediatric PC involvement in the ICU extend beyond family and patient support. Pediatric PC providers can assist the medical team with conflict resolution, helping to ease the moral distress often experienced during challenging cases and may also alleviate confusion surrounding prognosis and suffering at the end of life [45]. Pediatric PC team members can also provide the time and resources to support a family or patient when the medical team may be otherwise occupied, so that the family does not feel rushed or abandoned.

### 2.7. Barriers to Utilization of Pediatric PC Services for Perinatal and Neonatal Patients with CHD

Most surveyed medical providers in the pediatric cardiac ICU agree that PC consultation is helpful to both patients and families. However, utilization of PC services for pediatric ICU patients—particularly younger children with CHD—remains low, at <15% for all admissions and <40% for neonates who expired [9,10,11,46]. PC consultation occurs most frequently for infants and children with single ventricle heart disease, VADs, and genetic syndromes [7,47,48,49].

In one survey, the most common indications for PC consultation for children with advanced heart disease were goals of care and psychosocial support. It is important to note that initial pediatric PC consultation for children with cardiac disease is most frequently initiated in the ICU under “crisis” conditions, despite prenatal diagnosis [8]. Indeed, with the exception of congenital cardiac surgeons, most medical providers perceive that PC consultation occurs too late.

The most consistently cited barriers to PC consultation by providers were parental/familial factors, including the perception that involvement of PC occurs only after all other options have been exhausted and the medical care team has “given up.” Evidently, there is a misconception amongst both non-PC providers and families that PC consultation is only useful for end-of-life issues and unfamiliarity with the range of services that can be provided by PC providers. PC teams may mitigate these deficiencies by educating both families and medical providers about their role and principles [8,48,49]. Other barriers to pediatric PC consultation include staffing, limited capacity of hospices to provide care for infants, children, and adolescents, and concerns that involvement of a PC team may introduce inconsistencies in the medical care plan [24,47,48,49,50]. Resource limitations and challenges with communication may be decreased in the model proposed by Moynihan et al., which describes training multidisciplinary cardiac ICU team members as PC champions who facilitate earlier incorporation of PC principles into the care of pediatric cardiac ICU patients [19].

### 2.8. Increased Need for Pediatric PC Services during the COVID-19 Pandemic

In the current era, the global COVID-19 pandemic has presented several novel and significant stressors for both medical teams and families, affecting many aspects of care. Namely, visitor restrictions, increased use of technology, and decreased face-to-face interactions have changed our communication landscape, while providers and families alike are facing catastrophic times of job insecurity, limited interaction with distant family members, serious illness, and death. Women in the perinatal period are uniquely impacted by the current pandemic, with pregnancy and childbirth occurring in difficult conditions [51,52]. Pregnant women are now often alone for prenatal sonograms and visits with specialty physicians. When faced with a critical fetal diagnosis, this lack of support along with fears over pregnancy loss and contracting serious illness has adversely affected maternal mental health [52]. These unique challenges highlight a broad need for early PC consultation, although their full impact remains to be studied.

## 3. Conclusions

In conclusion, neonatal and perinatal CHD affect a large group of patients and families, with complex emotional, psychological, and medical needs that have likely expanded due to the current COVID-19 pandemic. While diagnostic and interventional capabilities have advanced substantially in recent decades, prognosis in infants with complex CHD remains uncertain, and the hospital course for individual patients is highly variable, requiring the expertise of multiple specialists. The pediatric PC team can assist the patient, family members, and medical teams throughout the process of learning the cardiac diagnosis as early as the prenatal period and navigating the healthcare system for the duration of the infant’s postnatal life. They can also substantially impact providers and families coping with moral distress, burnout, and communication challenges in a culturally sensitive manner. Empowering non-PC providers with basic PC skills, providing education to families and providers about the role of the PC team, and facilitating early engagement of subspecialty PC teams even at the time of fetal diagnosis may vastly improve the overall care of families and infants with CHD.

*“Endings matter, not just for the person but, perhaps even more, for the ones left behind.”*

*—Atul Gawande. Being Mortal: Medicine and What Matters in the End*

## Figures and Tables

**Table 1:** Common psychological, emotional, and cognitive transitions in families after a life-limiting pediatric cardiac diagnosis and targeted goals for pediatric PC involvement *.

Transition	Pediatric PC Involvement
Learning the diagnosis prenatally	“Big picture” discussions and psychosocial support with the likelihood of a poor outcome
Learning the diagnosis postnatally	Psychosocial support Increased parent education Continuity of care
New normal	Pain and symptom management Decision making support Care coordination Expanded support network with peer and emotional support
Taking control	Discussion regarding quality of life Respite care Advanced care planning
Learning death is likely	Anticipatory guidance Transition to hospice care Memory making
After death	Bereavement support Social and Emotional Support

* Adapted from Neubauer et al. [12].
